# Supplementary material for: Method for quantifying the Pasteurella multocida antigen adsorbed on aluminum hydroxide adjuvant in swine atrophic rhinitis vaccine
Source: PLoS One. 2024 May 20;19(5):e0301688. doi: 10.1371/journal.pone.0301688 (PMC11104628; doi:10.1371/journal.pone.0301688)
Supplement: S1 Table — (DOCX) [file pone.0301688.s001.docx]

Table S1. The raw data of Figure 1

|  | 1 | 2 | 3 | 4 | 5 | 6 |
| --- | --- | --- | --- | --- | --- | --- |
|  | 13336.94492 | 10052.21472 | 3027.597 | 12830.73 | 10410.02916 | 4832.3865 |
|  | 12839.12619 | 9867.991504 | 2373.924 | 13050.09 | 10141.69875 | 3965.610408 |
|  | 15015.0501 | 12461.64132 | 4659.002 | 15157.24 | 11165.02321 | 4789.566523 |
| Ave. | 13730.37373 | 10793.94918 | 3353.507753 | 13679.3547 | 10572.25037 | 4529.187811 |
| SD | 1140.066178 | 1447.198104 | 1176.885 | 1284.58 | 530.5987504 | 488.5417115 |
| SE | 658.2175149 | 835.540215 | 679.475 | 741.6529 | 306.3413314 | 282.0596887 |

Unpaired t-test

|  | 1 vs 4 | 2 vs 5 | 3 vs 6 |
| --- | --- | --- | --- |
| *P*-value | 0.961433414 | 0.815537118 | 0.185272449 |

1: PMT-alum prepared from 500 µg/mL PMT

2: PMT-alum prepared from 100 µg/mL PMT

3: PMT-alum prepared from 500 µg/mL PMT

4: Same sample as sample 1

5: Dilution sample 4 for 5 times with 5 mg/mL aluminum hydroxide

6: Dilution sample 4 for 50 times with 5 mg/mL aluminum hydroxide
