# Supplementary material for: Method for quantifying the Pasteurella multocida antigen adsorbed on aluminum hydroxide adjuvant in swine atrophic rhinitis vaccine
Source: PLoS One. 2024 May 20;19(5):e0301688. doi: 10.1371/journal.pone.0301688 (PMC11104628; doi:10.1371/journal.pone.0301688)
Supplement: S2 Table — (DOCX) [file pone.0301688.s002.docx]

Table S2. The raw data of Figure 2

|  | 1 | 2 | 3 | 4 | 5 | 6 | 7 | 8 |
| --- | --- | --- | --- | --- | --- | --- | --- | --- |
|  | 17285.23878 | 17581.73063 | 16710.01564 | 15580.26163 | 879.4943552 | 1348.117647 | 5699.965467 | 10698.40711 |
|  | 18969.31686 | 17345.60035 | 16199.64287 | 14632.23707 | 479.7236408 | 725.4032607 | 5430.17528 | 8657.134998 |
|  | 19663.45059 | 19399.37784 | 18063.94196 | 17716.62572 | 677.6687178 | 2851.500288 | 6323.032631 | 8779.135439 |
| Ave. | 18639.33541 | 18108.90294 | 16991.20016 | 15976.37481 | 678.962238 | 1641.673732 | 5817.72446 | 9378.225849 |
| SD | 1222.96307 | 1123.803133 | 963.4320558 | 1579.886889 | 199.8884962 | 1093.024942 | 457.9289765 | 1144.936657 |
| SE | 706.0780575 | 648.8280412 | 556.2377567 | 912.1481205 | 115.4056771 | 631.0582445 | 264.3854179 | 661.0294871 |

1: PMT-alum prepared from 10 mg/mL aluminum hydroxide

2: PMT-alum prepared from 7.5 mg/mL aluminum hydroxide

3: PMT-alum prepared from 5 mg/mL aluminum hydroxide

4: PMT-alum prepared from 2.5 mg/mL aluminum hydroxide

5: The collected supernatant from sample 1 before fixation

6: The collected supernatant from sample 2 before fixation

7: The collected supernatant from sample 3 before fixation

8: The collected supernatant from sample 4 before fixation
