# Supplementary material for: Method for quantifying the Pasteurella multocida antigen adsorbed on aluminum hydroxide adjuvant in swine atrophic rhinitis vaccine
Source: PLoS One. 2024 May 20;19(5):e0301688. doi: 10.1371/journal.pone.0301688 (PMC11104628; doi:10.1371/journal.pone.0301688)
Supplement: S3 Table — (DOCX) [file pone.0301688.s003.docx]

Table S3. The raw data of Table 1

|  | Std |
| --- | --- |
| BSA Conc. | Intensity |
| 400 | 11097.57998 |
| 200 | 6317.959566 |
| 100 | 3791.519678 |
| 50 | 1866.665438 |


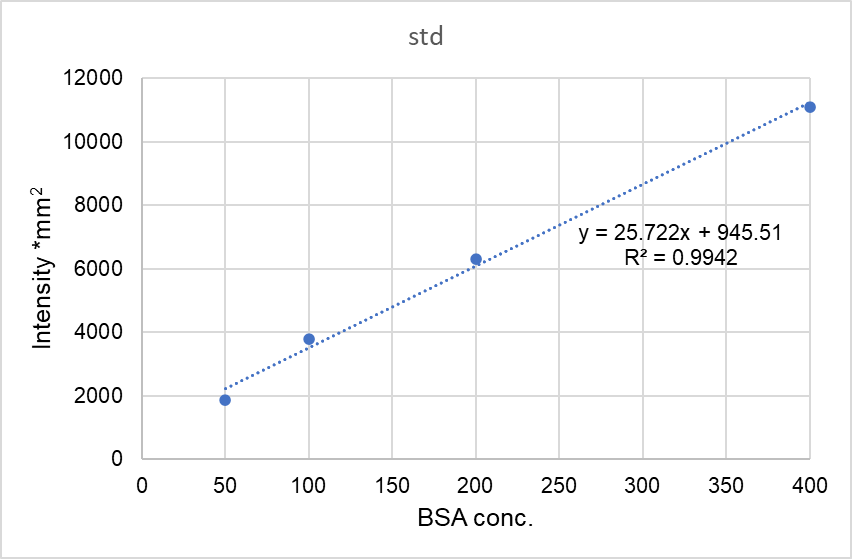


|  | The desorbed PMT samples | | | | | | | | | |
| --- | --- | --- | --- | --- | --- | --- | --- | --- | --- | --- |
|  | 1 | 2 | 3 | 4 | 5 | 6 | 7 | 8 | 9 | 10 |
| Theoretical value  (µg/mL) | 200 | 160 | 100 | 80 | 50 | 40 | 25 | 20 | 10 | 5 |
| Measured intensity | 2587.827078 | 2227.678151 | 1253.710849 | 1112.442279 | 869.378152 | 559.1426692 | - | - | - | - |
| Calculated concentration  (µg/mL) | 63.84808 | 49.84661 | 11.98182 | 6.489736 | -2.95984 | -15.0208 |  |  |  |  |
| Recovery rate  (%) | 31.92404 | 31.15413 | 11.98182 | 8.112169 |  |  |  |  |  |  |
