# Supplementary material for: Method for quantifying the Pasteurella multocida antigen adsorbed on aluminum hydroxide adjuvant in swine atrophic rhinitis vaccine
Source: PLoS One. 2024 May 20;19(5):e0301688. doi: 10.1371/journal.pone.0301688 (PMC11104628; doi:10.1371/journal.pone.0301688)
Supplement: S4 Table — The validation analysis for Table 2 is included. (DOCX) [file pone.0301688.s004.docx]

Table S4. The raw data of Table 2 and 3

1^st^

|  | standard | | | | | | | | | |
| --- | --- | --- | --- | --- | --- | --- | --- | --- | --- | --- |
|  | 200 | 160 | 100 | 80 | 50 | 40 | 25 | 20 | 10 | 5 |
|  | 5749.974821 | 4585.599887 | 3076.475476 | 2286.215764 | 1376.145268 | 1139.199951 | - | 882.5720411 | 736.6435048 | 946.4857232 |
|  | 5992.759227 | 4647.761842 | 3169.450785 | 2579.702915 | 1810.634226 | 1368.992386 | - | 1095.723076 | 1026.469061 | 1330.344923 |
| Ave. | 5871.367024 | 4616.680865 | 3122.96313 | 2432.959339 | 1593.389747 | 1254.096168 | - | 989.1475585 | 881.5562831 | 1138.415323 |

|  | 50 µg/mL  PMT-alum | AR Lot A | AR Lot B | AR/ER Lot C | AR/ER Lot D | Blank well |
| --- | --- | --- | --- | --- | --- | --- |
|  | 2629.773093 | 1275.749452 | 2333.378563 | 2735.533571 | 3419.76518 | -133.4049095 |
|  | 2080.582554 | 1349.540924 | 2425.550997 | 2803.400802 | 3286.633978 | -171.8820652 |
|  | 1933.656507 | 1560.587455 | 2412.425214 | 3305.78132 | 3984.660432 | 83.89431068 |
| Ave. | 2214.670718 | 1395.29261 | 2390.451591 | 2948.238565 | 3563.68653 | -73.79755467 |

| Slope | 25.76203701 | Intercept | 529.8689811 | R^2 | 0.9823 |  |
| --- | --- | --- | --- | --- | --- | --- |
|  | 50 µg/mL  PMT-alum | AR Lot A | AR Lot B | AR/ER Lot C | AR/ER Lot D | Blank well |
| Conc. (µg/mL) | 81.51157114 | 28.95269776 | 70.00648206 | 85.61685513 | 112.176541 | -25.74617412 |
|  | 60.19374838 | 31.81704705 | 73.58432158 | 88.25124429 | 107.0088129 | -27.23973443 |
|  | 54.49054847 | 40.00919933 | 73.07482061 | 107.7520515 | 134.1039705 | -17.31131239 |
| Ave. | 65.39862266 | 33.59298138 | 72.22187475 | 93.87338365 | 117.7631081 | -23.43240698 |

2^nd^

|  | standard | | | | | | | | | |
| --- | --- | --- | --- | --- | --- | --- | --- | --- | --- | --- |
|  | 200 | 160 | 100 | 80 | 50 | 40 | 25 | 20 | 10 | 5 |
|  | 10875.41662 | 9428.137885 | 7026.971162 | 6064.798973 | 4676.446361 | 3813.976597 | 2957.114789 | 1415.12118 | 1002.577461 | 836.0418101 |
|  | 10716.31365 | 9169.746084 | 6210.277413 | 5618.023601 | 3784.793322 | 3251.623781 | 1143.907715 | 1009.073446 | 540.0365521 | 405.0927997 |
| Ave. | 10795.86513 | 9298.941984 | 6618.624287 | 5841.411287 | 4230.619842 | 3532.800189 | 2050.511252 | 1212.097313 | 771.3070063 | 620.5673049 |

|  | 50 µg/mL  PMT-alum | AR Lot A | AR Lot B | AR/ER Lot C | AR/ER Lot D | Blank well |
| --- | --- | --- | --- | --- | --- | --- |
|  | 4469.292556 | 3815.047096 | 4697.527901 | 4956.686082 | 5694.576476 | 23.59356421 |
|  | 3996.740045 | 4100.335195 | 5289.976349 | 4775.260758 | 5949.975744 | -2.074092652 |
|  | 3806.093828 | 3562.713349 | 5102.833584 | 5422.146535 | 6329.857065 | -145.35071 |
| Ave. | 4090.70881 | 3826.03188 | 5030.112612 | 5051.364458 | 5991.469762 | -41.27707947 |

| Slope | 53.28514792 | Intercept | 820.5993535 | R^2 | 0.9725 |  |
| --- | --- | --- | --- | --- | --- | --- |
|  | 50 µg/mL  PMT-alum | AR Lot A | AR Lot B | AR/ER Lot C | AR/ER Lot D | Blank well |
| Conc. (µg/mL) | 68.47486298 | 56.19666754 | 72.75814555 | 77.6217556 | 91.46971177 | -14.95737218 |
|  | 59.60649103 | 61.5506566 | 83.8765992 | 74.21695461 | 96.26277848 | -15.43907596 |
|  | 56.02864196 | 51.46113134 | 80.36449927 | 86.3570312 | 103.3919943 | -18.12794186 |
| Ave. | 61.36999866 | 56.40281849 | 78.99974801 | 79.39858047 | 97.04149486 | -16.17479667 |

3^rd^

|  | standard | | | | | | | | | |
| --- | --- | --- | --- | --- | --- | --- | --- | --- | --- | --- |
|  | 200 | 160 | 100 | 80 | 50 | 40 | 25 | 20 | 10 | 5 |
|  | - | 8437.664367 | 6549.680474 | 5530.589344 | 3928.015198 | 3164.116534 | 1810.421342 | 1711.290662 | 1112.638183 | 1197.304957 |
|  | - | 8559.79862 | 6529.86407 | 5796.146192 | 4127.115928 | 3429.077309 | 2149.623685 | 1999.060373 | 1569.595464 | 1396.697641 |
| Ave. | - | 8498.731493 | 6539.772272 | 5663.367768 | 4027.565563 | 3296.596922 | 1980.022514 | 1855.175517 | 1341.116824 | 1297.001299 |

|  | 50 µg/mL  PMT-alum | AR Lot A | AR Lot B | AR/ER Lot C | AR/ER Lot D | Blank well |
| --- | --- | --- | --- | --- | --- | --- |
|  | 4522.835775 | 3267.054787 | 5024.36476 | 5361.170644 | 5986.062523 | -53.37899446 |
|  | 4016.927475 | 3303.768051 | 5159.734279 | 5072.47641 | 5925.700953 | -72.84262051 |
|  | 4010.601796 | 3604.043142 | 5343.945335 | 5104.445416 | 7391.251171 | -59.6073548 |
| Ave. | 4183.455015 | 3391.621994 | 5176.014791 | 5179.364157 | 6434.338215 | -61.94298992 |

| Slope | 49.98430046 | Intercept | 1111.893661 | R^2 | 0.9742 |  |
| --- | --- | --- | --- | --- | --- | --- |
|  | 50 µg/mL  PMT-alum | AR Lot A | AR Lot B | AR/ER Lot C | AR/ER Lot D | Blank well |
| Conc. (µg/mL) | 68.2402691 | 43.11676079 | 78.27399929 | 85.01223272 | 97.51399574 | -23.3127731 |
|  | 58.11892509 | 43.85125671 | 80.98224004 | 79.23653453 | 96.30638516 | -23.70216789 |
|  | 57.99237178 | 49.85864479 | 84.66761833 | 79.87611547 | 125.6265958 | -23.43737943 |
| Ave. | 61.45052199 | 45.60888743 | 81.30795255 | 81.37496091 | 106.4823256 | -23.4841068 |

Analysis for validation

Recovery rate

|  | Theoretical value (µg/mL) | Determined value (µg/mL) | Recovery rate (%) | Ave. | SD | Ave. ± 2SD | | |
| --- | --- | --- | --- | --- | --- | --- | --- | --- |
| 1^st^ | 50 | 61.37 | 122.74 | 125.48 | 4.61 | 116.27 | - | 134.69 |
| 2^nd^ | 50 | 65.40 | 130.80 |  |  |  |  |  |
| 3^rd^ | 50 | 61.45 | 122.90 |  |  |  |  |  |

Limit of quantification

|  | SD of blank value(δ) | Slope（S） | LOQ (10δ/S) | Ave. | SD |
| --- | --- | --- | --- | --- | --- |
| 1^st^ | 1.7085350 | 53.28514792 | 0.321 | 0.81 | 1.104653937 |
| 2^nd^ | 5.353366215 | 25.76203701 | 2.078 |  |  |
| 3^rd^ | 0.198858394 | 49.98430046 | 0.040 |  |  |

Linearity

|  | R^2 | Ave. | SD | Ave. ± 2SD | | |
| --- | --- | --- | --- | --- | --- | --- |
| 1^st^ | 0.9725 | 0.9763 | 0.00524 | 0.9658 | - | 0.9868 |
| 2^nd^ | 0.9823 |  |  |  |  |  |
| 3^rd^ | 0.9742 |  |  |  |  |  |

Precision

| N | J | | |  |  |
| --- | --- | --- | --- | --- | --- |
| 1 | 68.5 | 81.5 | 68.2 |  |  |
| 2 | 59.6 | 60.2 | 58.1 |  |  |
| 3 | 56.0 | 54.5 | 58.0 |  |  |
| TJ | T1 | T2 | T3 | TT | TT/JN |
|  | 184.110 | 196.196 | 184.352 | 564.657 | 62.740 |
|  | XJN^2 |  |  |  |  |
|  | 4688.8 | 6644.136 | 4656.734 |  |  |
|  | 3552.934 | 3623.287 | 3377.809 |  |  |
|  | 3139.209 | 2969.220 | 3363.115 | ΣΣXjn^2 |  |
| SUM | 11380.949 | 13236.643 | 11397.659 | 36015.252 |  |
|  |  |  |  |  |  |
| TJ^2 | 33896.491 | 38492.819 | 33985.500 | Σ（Tj^2/N） |  |
| TJ^2/N | 11298.830 | 12830.940 | 11328.500 | 35458.270 |  |

| N= |  | 3 |  | | |  |  |
| --- | --- | --- | --- | --- | --- | --- | --- |
| J= |  | 3 |  | | |  |  |
| CT= | TT^2/JN | 35426.446 |  | | |  |  |
| ST= | ΣΣXjn^2-CT | 588.806 |  | | |  |  |
| SRW= | Σ（Tj^2/N）-CT | 31.824 |  | | |  |  |
| Sr= | ST-SRW | 556.982 |  | | |  |  |
| φT= | JN-1 | 8 |  | | |  |  |
| φRW= | J-1 | 2 |  | | |  |  |
| φr= | J(N-1) | 6 |  | | |  |  |
| VRW= | SRW/φRW | 15.912 |  | | |  |  |
| Vr= | Sr/φr | 92.830 |  | | |  |  |
| Sr= | √Vr | 9.635 | : Repeatability / SD | | | CV(%)= | 15.356861 |
| Sa^2= | (VRW-Vr)/N | (25.639) |  | | |  |  |
| SIM^2= | Sr^2+Sa^2 | 67.191 |  | | |  |  |
| SIM= | √(Sr^2+Sa^2) | 8.197 | : Intermediate Precision / SD | | | CV(%)= | 13.065095 |
| φIM= | N^2SIM^4/(VRW^2/φRW+(N-1)^2Vr^2/φr) | 6.920 |  | | |  | |
| A= | VRW/JN | 1.768 |  | | |  |  |
| φA= | φRW | 2 |  | | |  |  |
|  |  |  |  | | |  |  |
|  |  |  |  | | |  |  |
| Confidence interval calculation (Intermediate Precision) | | | | | | | |
| φIMSIM^2 |  | 403.145 |  | | |  |  |
| χ^2(φIM,α/2) |  | 14.449 |  | | |  |  |
| χ^2(φIM,1-α/2) |  | 1.237 |  | | |  |  |
| Lower | =φIMSIM^2/χ^2(φIM,α/2) | 27.901 |  | | |  |  |
| Upper | =φIMSIM^2/χ^2(φIM,1-α/2) | 325.815 |  | | |  |  |
|  | =√(φIMSIM^2/χ^2(φIM,α/2)) | 5.282 |  |  |  |  |  |
|  | =√(φIMSIM^2/χ^2(φIM,1-α/2)) | 18.050 | : Confidence interval for standard deviation | | | | |
|  |  |  |  | | |  |  |
| Confidence interval calculation (Repeatability) | | | | | | | |
| φrSr^2 |  | 556.982 |  | | |  |  |
| χ^2(φr,α/2) |  | 14.449 |  | | |  |  |
| χ^2(φr,1-α/2) |  | 1.237 |  | | |  |  |
| Lower | =φrSr^2/χ^2(φr,α/2) | 38.547 |  | | |  |  |
| Upper | =φrSr^2/χ^2(φr,1-α/2) | 450.143 |  | | |  |  |
|  | =√(φrSr^2/χ^2(φr,α/2)) | 6.209 |  |  |  |  |  |
|  | =√(φrSr^2/χ^2(φr,1-α/2)) | 21.217 | : Confidence interval for standard deviation | | | | |

Calculation the average of PMT content of AR and AR/ER vaccines

| Conc. (µg/mL) | AR LotA | AR LotB | AR/ER Lot C | AR/ER Lot D |
| --- | --- | --- | --- | --- |
|  | 56.40281849 | 78.99974801 | 79.39858047 | 97.04149486 |
|  | 33.59298138 | 72.22187475 | 93.87338365 | 117.7631081 |
|  | 45.60888743 | 81.30795255 | 81.37496091 | 106.4823256 |
| Ave. | 45.20156243 | 77.50985844 | 84.88230834 | 107.0956428 |
