# Supplementary material for: Method for quantifying the Pasteurella multocida antigen adsorbed on aluminum hydroxide adjuvant in swine atrophic rhinitis vaccine
Source: PLoS One. 2024 May 20;19(5):e0301688. doi: 10.1371/journal.pone.0301688 (PMC11104628; doi:10.1371/journal.pone.0301688)
Supplement: S5 Table — Individual neutralizing antibody titer following vaccination is described. (DOCX) [file pone.0301688.s005.docx]

Table S5. Individual neutralizing antibody titer following vaccination

|  | AR 001 | AR 003 | ARER 003 | ARER 005 |
| --- | --- | --- | --- | --- |
|  | 64 | 16 | 8 | 64 |
|  | <2 | 64 | 16 | 8 |
|  | 8 | 16 | 32 | 16 |
|  | <2 | <2 | 32 | 32 |
|  | 4 | 32 | <2 | 32 |
|  | 4 | 32 | 32 | 8 |
|  | 4 | 8 | 128 | 16 |
|  | 8 | 16 | 32 | 4 |
|  | <2 | 32 | 4 | 4 |
|  | 4 | 16 | 8 | 16 |
| GMT | 4 | 16 | 14.928528 | 13.928809 |
| SD | 19.18014019 | 17.7266278 | 36.9625937 | 18.47520861 |
| SE | 6.065292885 | 5.60565191 | 11.68859843 | 5.842373947 |
| 95 CI | 11.88797405 | 10.98707774 | 22.90965293 | 11.45105294 |

For the calculation of the GMT, data reported as “<2” were converted to “1”. For the unpaired t-test between “AR 001 and AR 003” and “AR 001 and ARER 005”, the *p*-values are “0.122086146 “ and “0.245955004“, respectively.
