# Supplementary material for: Method for quantifying the Pasteurella multocida antigen adsorbed on aluminum hydroxide adjuvant in swine atrophic rhinitis vaccine
Source: PLoS One. 2024 May 20;19(5):e0301688. doi: 10.1371/journal.pone.0301688 (PMC11104628; doi:10.1371/journal.pone.0301688)
Supplement: S1 Fig — The MAb, which used in this study, did not react with the other antigens in the vaccine. (DOCX) [file pone.0301688.s006.docx]

Figure S1. Confirmation of MAb specificity

1 2 3 4 5 6


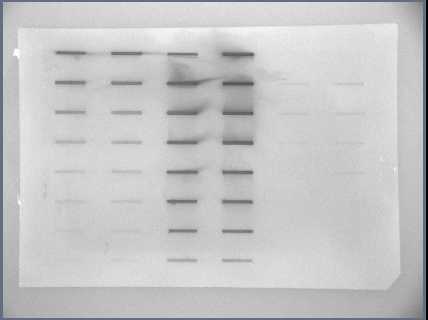


1: AR vaccine

2: AR/ER vaccine

3: purified PMT

4: PMT-alum

5: the dermonecrotic toxin of *B. bronchiseptica* conjugated with alum

6: the surface-protective antigen type A of *E. rhusiopathiae* conjugated with alum
