# Supplementary material for: Method for quantifying the Pasteurella multocida antigen adsorbed on aluminum hydroxide adjuvant in swine atrophic rhinitis vaccine
Source: PLoS One. 2024 May 20;19(5):e0301688. doi: 10.1371/journal.pone.0301688 (PMC11104628; doi:10.1371/journal.pone.0301688)
Supplement: Attachment — Submitted filename: PONE-D-23-12736_reviewer.pdf [file pone.0301688.s007.pdf]

PLOS ONE

Method for quantifying the Pasteurella multocida antigen adsorbed on aluminum hydroxide adjuvant in swine atrophic rhinitis vaccine

--Manuscript Draft--

**Manuscript Number:**	PONE-D-23-12736
**Article Type:**	Research Article
**Full Title:**	Method for quantifying the Pasteurella multocida antigen adsorbed on aluminum hydroxide adjuvant in swine atrophic rhinitis vaccine
**Short Title:**	Quantification of Pasteurella multocida antigen in AR vaccine
**Corresponding Author:**	Masaya Yoshimura, D.V.M<br>Meiji Animal Health Co., Ltd.<br>Kumamoto, Kumamoto JAPAN
**Keywords:**	Swine atrophic rhinitis; Pasteurella multocida; vaccine; Dot immunoassay; monoclonal antibody
**Abstract:**	Swine atrophic rhinitis is a disease caused by Pasteurella multocida and Bordetella bronchiseptica that affects pigs. Inactivated vaccines containing the toxins produced by Pasteurella multocida and Bordetella bronchiseptica have been widely used for the prevention of swine atrophic rhinitis. The efficacy of a vaccine is correlated with the amount of antigen present; however, the dermonecrotic toxin of P. multocida bound to aluminum hydroxide, which is used as an adjuvant, can hinder the monitoring of the antigen concentration in the vaccine. This study assessed the applicability of a dot immunoassay as an antigen quantification method using monoclonal antibodies. This quantification method was able to detect the antigen with high specificity and sensitivity even when the antigen was bound to the adjuvant, and its application to vaccine products revealed a correlation between the amount of antigen present in the vaccine and the neutralizing antibody titers induced in pigs. The antigen quantification method presented in this study is a simple and sensitive assay capable of quantifying the amount of antigen present in a vaccine that can be used as an alternative quality control measure.
**Order of Authors:**	Masaya Yoshimura, D.V.M
	Youko Honda
	Emi Yonemitsu
	Kasumi Kuraoka
	Kiyotaka Suenaga, D.V.M
**Additional Information:**	
**Question**	**Response**
**Financial Disclosure**<br><br>Enter a financial disclosure statement that describes the sources of funding for the work included in this submission. Review the submission guidelines for detailed requirements. View published research articles from *PLOS ONE* for specific examples.<br><br>This statement is required for submission and **will appear in the published article** if the submission is accepted. Please make sure it is accurate.	The authors received no specific funding for this work.

Powered by Editorial Manager® and ProduXion Manager® from Aries Systems Corporation

**Unfunded studies**<br>Enter: *The author(s) received no specific funding for this work.*<br><br>**Funded studies**<br>Enter a statement with the following details:<br>• Initials of the authors who received each award<br>• Grant numbers awarded to each author<br>• The full name of each funder<br>• URL of each funder website<br>• Did the sponsors or funders play any role in the study design, data collection and analysis, decision to publish, or preparation of the manuscript?<br>• **NO** - Include this sentence at the end of your statement: *The funders had no role in study design, data collection and analysis, decision to publish, or preparation of the manuscript.*<br>• **YES** - Specify the role(s) played.<br><br>* typeset	
**Competing Interests**<br><br>Use the instructions below to enter a competing interest statement for this submission. On behalf of all authors, disclose any competing interests that could be perceived to bias this work—acknowledging all financial support and any other relevant financial or non-financial competing interests.<br><br>This statement is required for submission and **will appear in the published article** if the submission is accepted. Please make sure it is accurate and that any funding sources listed in your Funding Information later in the submission form are also declared in your Financial Disclosure statement.<br><br>View published research articles from *PLOS ONE* for specific examples.	The authors have declared that no competing interests exist.

**NO authors have competing interests**<br><br>Enter: *The authors have declared that no competing interests exist.*<br><br>**Authors with competing interests**<br><br>Enter competing interest details beginning with this statement:<br><br>*I have read the journal's policy and the authors of this manuscript have the following competing interests: [insert competing interests here]*<br><br>* typeset	
**Ethics Statement**<br><br>Enter an ethics statement for this submission. This statement is required if the study involved:<br><br>• Human participants<br>• Human specimens or tissue<br>• Vertebrate animals or cephalopods<br>• Vertebrate embryos or tissues<br>• Field research<br><br>Write "N/A" if the submission does not require an ethics statement.<br><br>General guidance is provided below. Consult the submission guidelines for detailed instructions. **Make sure that all information entered here is included in the Methods section of the manuscript.**	The animal study was approved by the internal committee of KM Biologics Co., Ltd. in accordance with the "Guidelines for Proper Conduct of Animal Experiments". The approval number is "R200401-2, B20-028".<br>At the end of the study, all pigs were euthanized with intravenous injection of pentobarbital sodium and exsanguination.

Powered by Editorial Manager® and ProduXion Manager® from Aries Systems Corporation

**Format for specific study types**<br><br>**Human Subject Research (involving human participants and/or tissue)**<br>• Give the name of the institutional review board or ethics committee that approved the study<br>• Include the approval number and/or a statement indicating approval of this research<br>• Indicate the form of consent obtained (written/oral) or the reason that consent was not obtained (e.g. the data were analyzed anonymously)<br><br>**Animal Research (involving vertebrate animals, embryos or tissues)**<br>• Provide the name of the Institutional Animal Care and Use Committee (IACUC) or other relevant ethics board that reviewed the study protocol, and indicate whether they approved this research or granted a formal waiver of ethical approval<br>• Include an approval number if one was obtained<br>• If the study involved *non-human primates*, add *additional details* about animal welfare and steps taken to ameliorate suffering<br>• If anesthesia, euthanasia, or any kind of animal sacrifice is part of the study, include briefly which substances and/or methods were applied<br><br>**Field Research**<br><br>Include the following details if this study involves the collection of plant, animal, or other materials from a natural setting:<br>• Field permit number<br>• Name of the institution or relevant body that granted permission	
**Data Availability**<br><br>Authors are required to make all data underlying the findings described fully available, without restriction, and from the time of publication. PLOS allows rare exceptions to address legal and ethical concerns. See the PLOS Data Policy and FAQ for detailed information.	Yes - all data are fully available without restriction

Powered by Editorial Manager® and ProduXion Manager® from Aries Systems Corporation

A Data Availability Statement describing where the data can be found is required at submission. Your answers to this question constitute the Data Availability Statement and **will be published in the article**, if accepted.<br><br>**Important:** Stating 'data available on request from the author' is not sufficient. If your data are only available upon request, select 'No' for the first question and explain your exceptional situation in the text box.<br><br>Do the authors confirm that all data underlying the findings described in their manuscript are fully available without restriction?	
**Describe where the data may be found in full sentences. If you are copying our sample text, replace any instances of XXX with the appropriate details.**<br><br>• If the data are **held or will be held in a public repository**, include URLs, accession numbers or DOIs. If this information will only be available after acceptance, indicate this by ticking the box below. For example: *All XXX files are available from the XXX database (accession number(s) XXX, XXX.*).<br>• If the data are all contained **within the manuscript and/or Supporting Information files**, enter the following: *All relevant data are within the manuscript and its Supporting Information files.*<br>• If neither of these applies but you are able to provide **details of access elsewhere**, with or without limitations, please do so. For example:<br><br>*Data cannot be shared publicly because of [XXX]. Data are available from the XXX Institutional Data Access / Ethics Committee (contact via XXX) for researchers who meet the criteria for access to confidential data.*<br><br>*The data underlying the results presented in the study are available from (include the name of the third party*	All relevant data are within the manuscript and its Supporting Information files.

and contact information or URL).<br>• This text is appropriate if the data are owned by a third party and authors do not have permission to share the data.<br><br>* typeset	
Additional data availability information:	

Powered by Editorial Manager® and ProduXion Manager® from Aries Systems Corporation

Manuscript Click here to access/download;Manuscript;Manuscript.docx

Method for quantifying the *Pasteurella multocida* antigen adsorbed on aluminum hydroxide adjuvant in swine atrophic rhinitis vaccine

Masaya Yoshimura*, Youko Honda, Emi Yonemitsu, Kasumi Kuraoka, Kiyotaka Suenaga

Meiji Animal Health Co., Ltd., 1-6-1 Okubo, Kita-ku, Kumamoto-shi, Japan

* Corresponding author

E-mail: yoshimura-ma@vet.meiji.com (MY)

Short title: Quantification of *Pasteurella multocida* antigen in AR vaccine

Abstract

Swine atrophic rhinitis is a disease caused by *Pasteurella multocida* and *Bordetella bronchiseptica* that affects pigs. Inactivated vaccines containing the toxins produced by *Pasteurella multocida* and *Bordetella bronchiseptica* have been widely used for the prevention of swine atrophic rhinitis. The efficacy of a vaccine is correlated with the amount of antigen present; however, the dermonecrotic toxin of *P. multocida* bound to aluminum hydroxide, which is used as an adjuvant, can hinder the monitoring of the antigen concentration in the vaccine. This study assessed the applicability of a dot immunoassay as an antigen quantification method using monoclonal antibodies. This quantification method was able to detect the antigen with high specificity and sensitivity even when the antigen was bound to the adjuvant, and its application to vaccine products revealed a correlation between the amount of antigen present in the vaccine and the neutralizing antibody titers induced in pigs. The antigen quantification method presented in this study is a simple and sensitive assay capable of quantifying the amount of antigen present in a vaccine that can be used as an alternative quality control measure.

Introduction

Swine atrophic rhinitis (AR), a common respiratory disease affecting pigs worldwide, is characterized by hypoplasia or atrophy of the nasal turbinates due to upper respiratory tract infections caused by *Pasteurella multocida* and *Bordetella bronchiseptica*. AR is rarely fatal; however, it is a threat to the swine industry as it is easily transmitted. Moreover, it impedes weight gain in affected pigs and reduces the production efficiency, resulting in economic losses. AR cannot be cured after it has progressed to the lesion stage. Therefore, formulation of preventive measures that combine improvement of the environmental conditions of the farm and a farm-based vaccination program is essential [1, 2].

The development of the upper respiratory lesions is likely triggered by infection with *B. bronchiseptica* or poor environmental conditions. *B. bronchiseptica* colonizes the nasal passages of pigs and induces nasal mucosal inflammation. In contrast, *P. multocida* cannot colonize normal nasal mucosa; however, it can colonize nasal mucosa that is damaged by primary factors [3, 4]. The toxins produced by *B. bronchiseptica* and *P. multocida* cause AR. In particular, *P. multocida* produces a toxin that causes progressive nasal turbinate atrophy [3, 5]. The onset of AR is marked by the manifestation of acute catarrhal rhinitis symptoms, such as sneezing and a runny nose. Inflammation of the nasolacrimal duct is commonly observed following infection with *P. multocida,* and it can result in ductal occlusion and subsequent lacrimation of the medial canthus. Facial deformities due to the delayed development of the nasal, maxillary, and frontal bones usually become apparent one

month after the onset of the disease. These clinical manifestations have been attributed to the mitogenic dermonecrotic toxins of *P. multocida* (PMT) [6]. Several vaccines have been developed for the prevention of AR. An attenuated *P. multocida* mutant expressing only the N-terminally truncated fragment of PMT that can increase antibody production has been used to prevent AR in pigs [7]. Vaccines produced using recombinant derivatives of PMT alone have also shown protective effects [8, 9]. Two bivalent and trivalent AR vaccines, Suimmugen rART$_2$ and Suimmugen rART$_2$/ER (Meiji Animal Health Co., Ltd., Japan), containing recombinant proteins expressed in *Escherichia coli* and aluminum hydroxide as an adjuvant have been available in Japan. Two antigens are present in these vaccines: the non-toxic derivatives of PMT and *B. bronchiseptica* dermonecrotic toxin. In addition, Suimmugen rART$_2$/ER contains the surface-protective antigen type A of *Erysipelothrix rhusiopathiae* to prevent swine erysipelas. As described by the approved application forms, these products can induce neutralizing antibodies and curb the action of PMT in vaccinated pigs.

Assays to evaluate the efficacy of these vaccine products are limited to in vivo testing, such as the immunization of pigs and mice. A previous study optimized the protein content of PMT in the vaccine dose to induce a protective antibody titer. Although the amount of PMT present in these vaccines is estimated using a physicochemical method during the vaccine manufacturing process, the amount of PMT bound to aluminum hydroxide, used as an adjuvant in the vaccine, remains

uncertain. Therefore, the present study used dot blotting and immunostaining assays to determine the amount of PMT-immunogenic antigens present in vaccine products.

Materials and methods

PMT

The PMT protein is expressed in the *E. coli* PRX-1 strain transfected with the plasmid pRPX-1 encoding the non-toxic PMT gene, *toxA-SQ*, of *P. multocida*, a toxin-producing pathogen. PMT was purified from the solubilized total protein via ion exchange chromatography using diethylaminoethyl -dextran and an elution buffer containing 0.5 M NaCl in 0.05 M phosphate buffer (pH 7.2) from the solubilized total protein of *E. coli*. The purified proteins were separated on a 5–20% w/v acrylamide gel using sodium dodecyl-sulfate polyacrylamide gel electrophoresis (SDS-PAGE), and their concentrations were compared with a bovine serum albumin standard using the GelDoc XR system (BIO-RAD; California 94547, USA) after staining with Coomassie blue R250.

Adsorption of PMT on aluminum hydroxide

PMT and aluminum hydroxide (ALHYDROGEL 85, CRODA Health Care. Shiga, Japan) were diluted with the elution buffer to attain arbitrary concentrations. Subsequently, the PMT solution was mixed with aluminum hydroxide and incubated at 4°C overnight with stirring. Thereafter, the

mixture was fixed with 10% formalin (FUJIFILM Wako Pure Chemical Corporation.; Osaka, Japan), N-acetyl-DL-tryptophan (Ajinomoto Healthy Supply Co., Inc.; Tokyo, Japan), and L-lysine hydrochloride (FUJIFILM Wako Pure Chemical Corporation.; Osaka, Japan) at 4°C overnight. Lastly, the aluminum hydroxide combined with PMT was washed with phosphate-buffered saline (PBS) and defined as PMT-alum. The samples were collected as a mixture of PMT and aluminum hydroxide supernatants before fixation and used for the detection of PMT that was not adsorbed on aluminum hydroxide.

Anti-PMT monoclonal antibody (MAb)

The antibody-producing hybridoma cell line, 1F3, was obtained (provided by Prof. Horiguchi of Osaka University). Murine ascites from BALB/c mice with a hybridoma transplant possess the ability to neutralize PMT; consequently, they have been used as a monoclonal antibody (Mab). The MAb immunoglobulin was purified via affinity chromatography using Protein G, followed by ammonium sulfate precipitation. Thereafter, the purified MAb were labeled with horseradish peroxidase (HRP) using a Peroxidase Labeling Kit-NH2 (Dojindo Laboratories; Kumamoto, Japan).

Dot blotting and immunostaining assay

All samples were diluted to a concentration of 1:400 with PBS containing 0.12 M NaCl in 0.05 M phosphate buffer (pH 6.8) before the assay. Subsequently, 200 µL of each sample was immobilized on a nitrocellulose membrane (Premium NC, 0.45; Amersham) using a suction blotter (Bio-Rad Laboratories). The membranes were soaked in 0.05% Tween 20 in PBS (T/PBS) containing 5% skim milk at room temperature (RT) for 1 h. After blocking the empty surface of the membrane, it was immersed in a 1:5000 dilution MAb in T/PBS and incubated at RT for 1 h with gentle shaking. After the first reaction, the membrane was soaked in T/PBS and washed (the membrane was washed between every reaction) and soaked in T/PBS containing 1:5000 dilution horseradish peroxidase-labelled anti-mouse IgG (Jackson ImmunoResearch; USA). Subsequently, it was incubated at RT for 1 h with gentle shaking. In addition to indirect staining, the membrane was soaked in a 1:5000 dilution of HRP-labeled MAb, followed by blotting and blocking, when HRP-labeled MAb was used for direct staining. After the final wash, the membrane was soaked in 10 mL of a color developer, Easy West Blue (ATTO; Tokyo, Japan), and incubated at RT for 5 min with gentle shaking. After color development, the membrane was washed with water to terminate the reaction. The color intensities of all dotted points were scanned using an image analyzer (GelDoc XR; Bio-Rad), and images were captured.

Serum neutralization test for the detection of anti-PMT dermonecrotic toxin

A microneutralization test was performed using a 96-well microplate to detect the neutralizing antibodies. *P. multocida* strain S70 was cultured in a heart infusion broth for 18 h at 37°C using a shake culture, and the grown cells were concentrated followed by disruption via sonication. The centrifuged supernatant was filtered using a 0.45 µm filter as a challenge Pm toxin. Pig sera were heated at 56℃ for 30 min before the assay. The sera and Pm toxin were diluted with supplemented Eagle's minimal essential medium (E-MEM; Nissui Pharmaceutical Co., Ltd.; Tokyo, Japan) containing trypticase soy broth (Becton, Dickinson and Company; Tokyo, Japan), sodium bicarbonate (FUJIFILM Wako Pure Chemical Corporation.; Osaka, Japan), L-glutamine (FUJIFILM Wako Pure Chemical Corporation.; Osaka, Japan), and gentamycin (FUJIFILM Wako Pure Chemical Corporation.; Osaka, Japan). Subsequently, 50 µL of serial duplicate two-fold dilutions was mixed with an equal volume of Pm toxin containing 80 units/mL and incubated at 37°C for 1 h. Thereafter, 100 µL of bovine embryonic lung cells, EBL cell (ACC 192) suspension (4×10^5 cells/mL) in E-MEM and Hanks (Nissui Pharmaceutical Co., Ltd.; Tokyo, Japan) mixture medium containing 10% FBS, sodium bicarbonate, L-glutamine, penicillin (Meiji Seika Pharma Co., Ltd.; Tokyo, Japan), streptomycin (Meiji Seika Pharma Co., Ltd.; Tokyo, Japan), and kanamycin (FUJIFILM Wako Pure Chemical Corporation.; Osaka, Japan) were added to the toxin-serum

mixtures and cultured at 37°C in 5% CO_2 for 7 days. The neutralizing antibody titer was expressed as the reciprocal of the highest serum dilution that inhibited the development of the cytopathic effect (CPE).

Serum collection from pigs inoculated with AR and AR/ER vaccine for the neutralization test

Ten 4-week-old pigs were vaccinated twice with two lots each of AR and AR/ER vaccines at a 4-week interval. The sera were collected 4 weeks after the last vaccination and used for the neutralization test. The sera of pigs vaccinated with AR vaccine Lot A and AR/ER vaccine Lot C were obtained from a field farm. The sera of pigs vaccinated with AR vaccine Lot B and AR/ER vaccine Lot D were obtained through animal experiments in an in-house animal facility.

Ethics statement

Twenty 3-month-old LWD crossbred pigs (Landrace, Large White, and Duroc) were used in the in-house animal study and acclimated for 7 days prior to the study. The pigs had free access to water and were fed a commercial diet (Nosan Corporation; Yokohama, Japan). Their health and behavior were monitored daily. The pigs were retained before immunization and blood collection. At the end of the study period, all pigs were pre-anesthetized with pentobarbital sodium and euthanized via

exsanguination by dissecting the axillary artery. Since approved and commercially available vaccines were used in this study, we hypothesized that there would be no deaths or serious adverse events during the study period; therefore, no humane endpoints were set. This study was approved by the internal committee of KM Biologics Co., Ltd., and conducted in accordance with the "Guidelines for Proper Conduct of Animal Experiments" (Protocol No.: R200401-2, Approval No.: B20-028). All experimenters and staff received annual training. Only registered individuals conducted animal experiments.

Results

Adsorption efficiency of PMT on aluminum hydroxide at different mixing ratios

Different concentrations of PMT and aluminum hydroxide were used to prepare PMT-alum. Subsequently, the amount of PMT in the final product was analyzed to determine the influence of the mixing ratio of PMT and aluminum hydroxide on the adsorption efficacy.

Different concentrations of PMT (500, 100, and 10 µg/mL) were mixed with an equal volume of 10 mg/mL of aluminum hydroxide to prepare PMT-alum to confirm the effect of PMT concentration on the PMT-alum preparation process. In addition, the PMT-alum prepared using 500 µg/mL PMT was diluted five and 50 times with 5 mg/mL aluminum hydroxide, which were assumed

to be 50 and 5 µg/mL of PMT in PMT-alum, respectively. The samples from each preparation were processed for dot blotting and indirect immunostaining. The results are presented in Fig 1 and Table S1. The dots immobilized with PMT-alum prepared from 500, 100, and 10 µg/mL of starting materials, which may be half the concentration in PMT-alum, showed different intensities according to the PMT concentration (Fig 1A). A similar result was obtained for the dot immunostaining assay of dilutions of the PMT-alum preparation (Fig 1B). No differences were observed between 50 and 5 µg/mL of the starting material PMT-alum (intensity of 7713.1 and 4049.0, respectively), which was prepared by diluting PMT-alum five and 50 times (intensity of 7615.0 and 3440.1, respectively). The differences between the former and latter samples were 1.27 and 15.0%, respectivel[illegible]

Fig 1. The intensities of the dot blotting spot of PMT-alum prepared from diluted PMT solutions compared with those of the dilutions of the PMT-alum sample. A) PMT dilution of 500, 100, and 10 µg/mL was mixed with an equal volume of 10 mg/mL aluminum hydroxide and integrated into PMT-alum. B) PMT concentrations were adjusted to 50 and 5 µg/mL PMT-alum via dilution at 1:5 and 1:50 of PMT-alum sample prepared from 500 µg/mL PMT.

PMT, toxins of *P. multocida;* conc, concentration.

Subsequently, different concentrations of aluminum hydroxide (10, 7.5, 5, and 2.5 mg/mL) were mixed with an equal volume of 500 μg/mL PMT to prepare PMT-alum to confirm the effect of the aluminum hydroxide concentration on the process. A portion of the supernatant was collected immediately before fixation to detect non-adsorbed PMT. The PMT-alum and supernatant of the mixtures were aligned and processed for dot blotting and immunostaining assays. The results are presented in Fig 2 and Table S2. The intensities of the PMT-alum samples prepared using different concentrations of aluminum hydroxide varied; however, their levels decreased with the reduction in the aluminum hydroxide concentration. The intensities of the PMT-alum samples prepared from aluminum hydroxide (10, 7.5, 5, and 2.5 mg) were 13703, 11979, 9880, and 8397, respectively. A positive reaction was observed at lower aluminum concentrations in the dot immunostaining assay of the supernatant of the PMT and aluminum hydroxide mixture. An apparent positive reaction was observed in the supernatant of the mixture at 2.5 mg/mL with an intensity of 4800, which was three times higher than that for the 5 mg/mL of aluminum used.

Fig 2. Intensities of the dot blotting spots of PMT-alum and the supernatants of the mixture before fixation. A constant concentration of PMT, 500 μg/mL, was mixed with an equal volume of aluminum hydroxide diluted to 10, 7.5, 5, and 2.5 mg/mL and integrated into PMT-alum. Before fixation, the supernatants were collected from the PMT and aluminum hydroxide mixtures.

The black bars indicate the intensities of the PMT-alum, and the white bars indicate those of the supernatants.

PMT, toxins of *P. multocida;* conc, concentration.

Quantitative dot-blotting and immunostaining assay

The abovementioned data indicate that 10 mg/mL of aluminum hydroxide could adsorb almost all of the 500 µg/mL PMT to form 250 µg/mL PMT-alum. Therefore, standard serial dilution ranging from 200 to 5 µg/mL of PMT-alum was prepared via the dilution of the 250 µg/mL PMT-alum stock. In this experiment, standard dilutions of 200, 160, 100, 80, 60, 40, 20, 10, and 5 µg/mL of PMT-alum were used to create a calibration line. Concurrently, indirect and direct immunostaining were also evaluated. As shown in Fig 3, the indirect and direct immunostaining methods showed an apparent colorimetric reaction and good linearity of intensities. Although no difference in the linearity was observed between the two methods (indirect method: r^2 = 0.9893; direct method: r^2 = 0.9935), the slope of the quantitative line was steeper for the direct method than that for the indirect method. HRP-labeled MAb was used for subsequent quantification assays. The row data are shown in Table S3.

Fig 3. Serial standard dilution ranging from 200 to 5 μg/mL PMT-alum was used in the dot immunoassay. Intensity curves of the indirect (left) and direct (right) immunostaining method.

PMT, toxins of *P. multocida;* conc, concentration.

PMT content and immunogenicity of the AR vaccine product

The abovementioned experiments demonstrated that dot blotting and direct immunostaining could detect and quantify PMT antigens adsorbed on the adjuvant in vaccines. The sera of pigs who had received commercial AR and AR/ER vaccines were collected to evaluate the correlation between the PMT content and antibody titers. Two samples collected from each of the AR and AR/ER vaccine groups were used in the established dot immunoassay. The PMT content was estimated by comparing its intensity with that of the standard line. The sera collected from pigs vaccinated with these four vaccine lots were tested for the presence of neutralizing antibodies against PMT. Table 1 presents the PMT content in the vaccine and the geometric mean antibody titer of every 10 serum samples. The PMT content in the vaccine products of AR Lot A, AR Lot B, AR/ER Lot C, and AR/ER Lot D was approximately 50, 80, 100, and 100 μg of PMT, respectively, and these vaccines elicited antibodies in pigs at geometric mean titers of 4.0, 16.0, 14.9, and 13.9, respectively. The individual data are shown in Table S4.

Table 1. The PMT content of AR and AR/ER vaccines and the neutralization antibody titer elicited by these vaccines

	AR Lot A	AR Lot B	AR/ER Lot C	AR/ER Lot D
**PMT content (μg/mL)**	49.713	81.062	102.38	102.80
**Antibody titer (GMT, n=10)**	4.0	16.0	14.9	13.9

AR, atrophic rhinitis; PMT, toxins of P. multocida; GMT, geometric mean titers

Discussion

Swine AR is caused by PMT [6], which induces the production of neutralizing antibodies in the host and alleviates the disease symptoms [1]. Thus, PMT is an essential component of AR vaccines for pigs, and these vaccines contain adequate concentrations of antigens for antibody induction. In principle, the efficacy of the vaccine, that is, its ability to induce antibodies, is correlated with the antigen dose. Antigen doses play a significant role in inactivated vaccines as they have no replication activity and lower prolonged antigen persistence than live vaccines [10]. Antigen concentration in a vaccine must be measured to evaluate the effects of inactivated vaccines and toxoids. However, as a method for quantifying the antigen bound to the aluminum adjuvant is yet to

be established, the antigen concentration in the final product is calculated based on the values measured during this process. Animal experiments are often used as an alternative approach for quantifying the antigen concentration in the final product to determine efficacy.

A dot blotting and immunostaining method was established to measure the concentration of PMT antigens bound to the aluminum hydroxide adjuvant in the present study. This method could quantify PMT in vaccine products with high specificity and sensitivity. Since the MAb used in this study can neutralize the dermonecrotic PMT, the antigen detected using this method is the neutralizing epitope of PMT. Therefore, the amount of antigen quantified using this method is equivalent to the concentration of PMT. This method can also detect antigens bound to aluminum hydroxide, indicating that AR vaccines can appropriately induce neutralizing antibodies against PMT. MAb did not react with the other antigens in the vaccine, such as the dermonecrotic toxin of *B. bronchiseptica* and the surface-protective antigen type A of *E. rhusiopathiae*.

Aluminum-containing adjuvants are widely used in commercial vaccines due to their safety and enhanced potency. Aluminum hydroxide has an isoelectric point of approximately 11 and adsorbs acidic proteins due to the presence of attractive electrostatic forces [11]. Ganfield et al. [12] reported that the protective antigens of *P. multocida* have precipitinogenic activity in the pH range of 3–4, with the activity peaking at a pH of 3.7. Therefore, PMT appeared to bind effectively to aluminum hydroxide. In the current vaccine production method, the antigen concentration measured by SDS-

PAGE is converted to a concentration after binding with aluminum hydroxide. This concentration is calculated by assuming that the antigen was adsorbed 100% by aluminum hydroxide; however, the adsorption efficiency was not confirmed. Therefore, various antigen and aluminum hydroxide concentrations were prepared, and their adsorption efficiencies were evaluated. The experimental simulation revealed that PMT could be detected in the supernatant as an inverse proportion of the concentration of aluminum hydroxide by assigning different concentrations of aluminum hydroxide and evaluating the binding ability with 500 µg/mL PMT. Since the concentration of PMT obtained during manufacturing is usually approximately 500 µg/mL, it was assumed that all antigens can be adsorbed by 10 mg/mL aluminum hydroxide. However, if the yield of PMT is exceedingly high, the actual amount of antigen in the product may be less than the value converted from the PMT concentration measured by SDS-PAGE, as the adsorption capacity of aluminum hydroxide will be exceeded (PMT will be saturated). It would be possible to closely monitor the concentration of the PMT antigen in the final product using this method, which could not be measured previously. In this study, the neutralizing antibodies elicited by the vaccines were correlated with the amount of PMT antigen in the vaccine products (Table 1), indicating that this in vitro method reflected the in vivo test results.

In conclusion, this method can be a valuable tool that can be used for quality control of vaccine consistency during production. This quantification method can be easily applied to other antigens if

suitable antibodies are available. Furthermore, there is an opportunity to reduce the number of test animals, such as mice, used in quality control examinations by replacing animal models, which is beneficial from an animal welfare perspective.

Acknowledgments

The authors are grateful to Prof. Yasuhiko Horiguchi for providing hybridomas. The authors also thank Dr. Tomoyuki Tsuda for their assistance in preparing this manuscript. We would like to thank Honyaku Center Inc. (www.honyakuctren.com) and Editage (www.editage.com) for English language editing.

References

1. Sakano T, Okada M, Taneda A, Mukai T, Sato S. Effect of *Bordetella bronchiseptica* and serotype D *Pasteurella multocida* bacterin-toxoid on the occurrence of atrophic rhinitis after experimental infection with *B. bronchiseptica* and toxigenic type A *P. multocida*. J Vet Med Sci. 1997; 59: 55-57. doi: 10.1292/jvms.59.55

2. Sawata A, Nakai T, Tuji M, Kume K. Dermonecrotic activity of *Pasteurella multocida* strains isolated from pigs in Japanese field. Nihon Juigaku Zasshi. 1984; 46: 141-148. doi: 10.1292/jvms1939.46.141

3. Helke KL, Ezell PC, Duran-Struuck R, Swindle MM. Biology and diseases of Swine American College of Laboratory Animal Medicine. In: Fox JG, Anderson LC, Otto GM, Pritchett-Corning KR, Whary MT, editors. Laboratory animal medicine. San Diego: Academic Press; 2015. pp. 695-769.

4. Hamilton TD, Roe JM, Hayes CM, Jones P, Pearson GR, Webster AJ. Contributory and exacerbating roles of gaseous ammonia and organic dust in the etiology of atrophic rhinitis. Clin Diagn Lab Immunol. 1999; 6: 199-203. doi:10.1128/CDLI.6.2.199-203.1999

5. Horiguchi Y. Swine atrophic rhinitis caused by *Pasteurella multocida* toxin and *Bordetella* dermonecrotic toxin. Curr Top Microbiol Immunol. 2012; 361: 113-129. doi: 10.1007/82_2012_206

6. Martineau-Doizé B, Dumas G, Larochelle R, Frantz JC, Martineau GP. Atrophic rhinitis caused by *Pasteurella multocida* type D: morphometric analysis. Can J Vet Res. 1991; 55: 224-228.

7. Kim T, Son C, Lee J, Kim K. Vaccine potential of an attenuated *Pasteurella multocida* that expresses only the N-terminal truncated fragment of *P. multocida* toxin in pigs. Can J Vet Res. 2012; 76: 69-71.
8. Wu MC, Lo YT, Wu HC, Wang HW, Chu CY. Cross-protection of recombinant *Pasteurella multocida* toxin proteins against atrophic rhinitis in mice. Res Vet Sci. 2021; 137: 138-143. doi: 10.1016/j.rvsc.2021.05.002
9. Petersen SK, Foged NT, Bording A, Nielsen JP, Riemann HK, Frandsen PL. Recombinant derivatives of *Pasteurella multocida* toxin: candidates for a vaccine against progressive atrophic rhinitis. Infect Immun. 1991; 59: 1387-1393. doi:10.1128/iai.59.4.1387-1393.1991
10. Plotkin SA, Orenstein WA, Offit PA. Vaccines. 6th ed. Saunders; 2013.
11. Rinella JV Jr, White JL, Hem SL. Treatment of aluminium hydroxide adjuvant to optimize the adsorption of basic proteins. Vaccine 1996; 14: 298-300. doi: 10.1016/0264-410x(95)00194-6
12. Ganfield DJ, Rebers PA, Heddleston KL. Immunogenic and toxic properties of a purified lipopolysaccharide-protein complex from *Pasteurella multocida*. Infect Immun. 1976; 14: 990-999. doi: 10.1128/iai.14.4.990-999.1976

Figure 1

Click here to access/download;Figure;Fig 1.tif

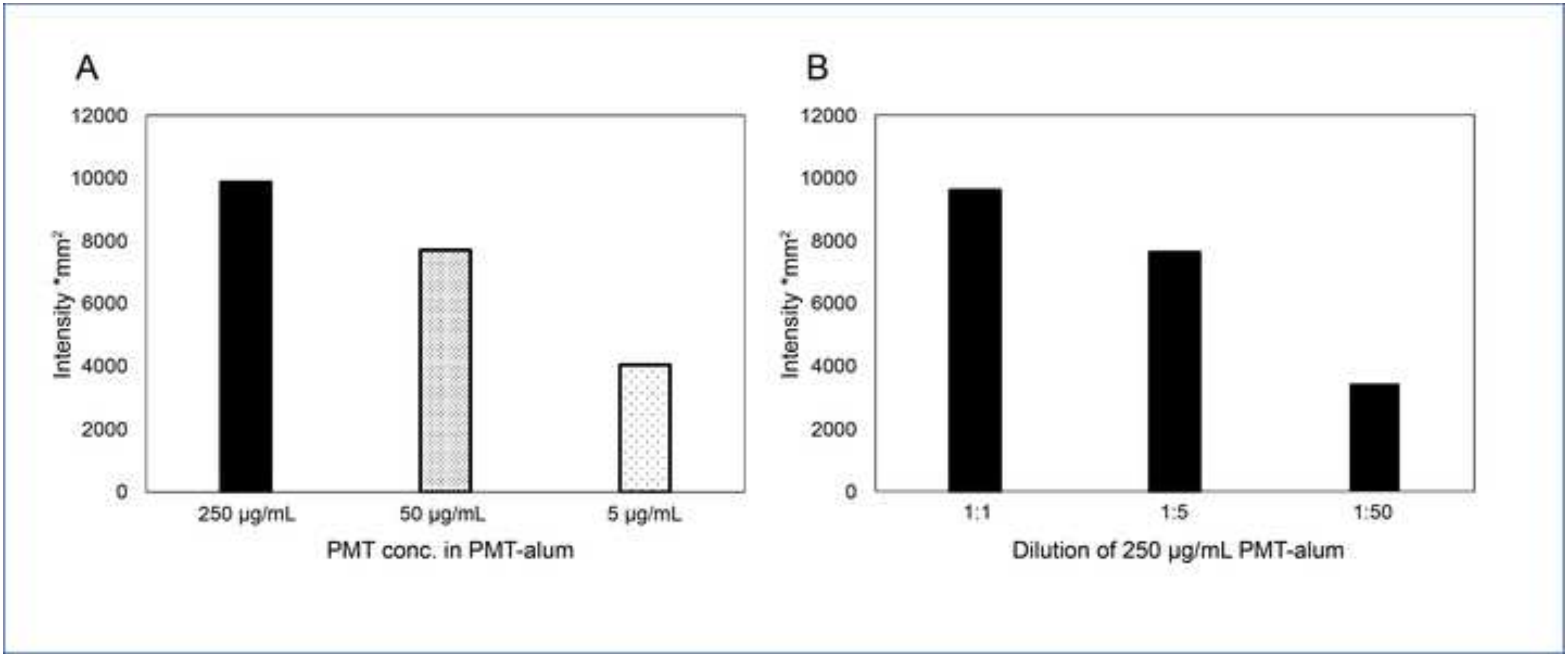

Figure 2

Click here to access/download;Figure;Fig 2.tif

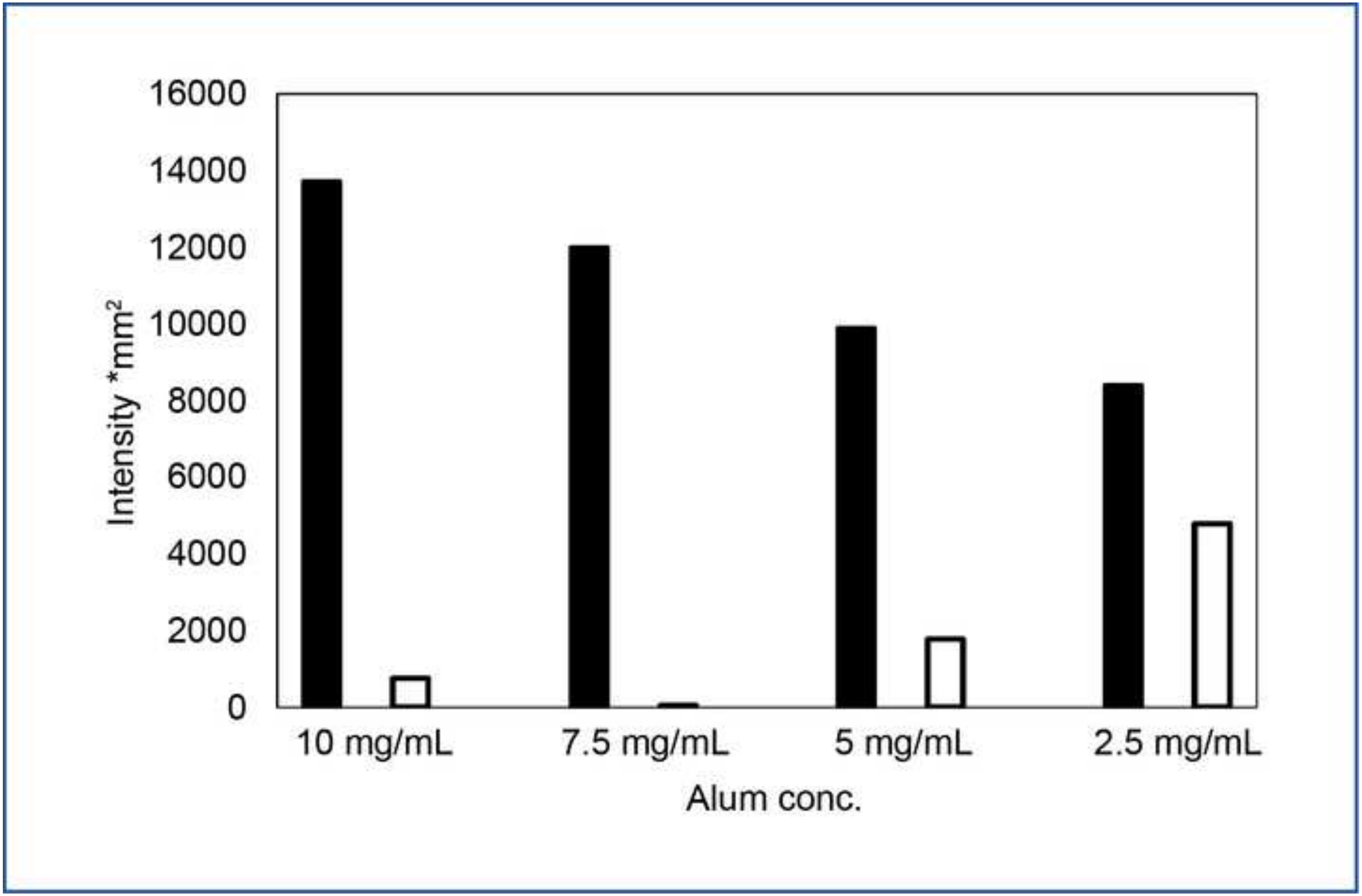

Figure 3

Click here to access/download;Figure;Fig 3.tif

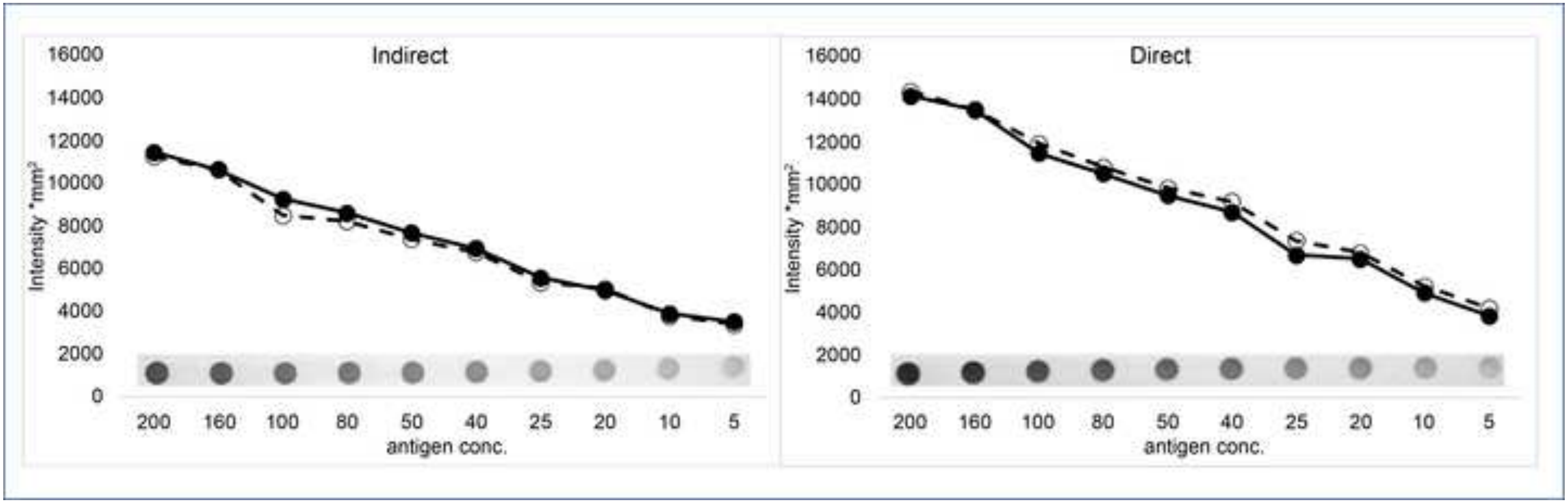

Table S1

Click here to access/download
Supporting Information
Table S1.docx

Table S2

Click here to access/download
Supporting Information
Table S2.docx

Table S3

Click here to access/download
Supporting Information
Table S3.docx

Table S4

Click here to access/download
Supporting Information
Table S4.docx
