# Supplementary material for: Method for quantifying the Pasteurella multocida antigen adsorbed on aluminum hydroxide adjuvant in swine atrophic rhinitis vaccine
Source: PLoS One. 2024 May 20;19(5):e0301688. doi: 10.1371/journal.pone.0301688 (PMC11104628; doi:10.1371/journal.pone.0301688)
Supplement: Attachment — Submitted filename: Reviewer#2.pdf [file pone.0301688.s009.pdf]

Response to Reviewers:
Thank you for your review of our paper. Our answers to your comments are as follows.

No.	Your comments	Our answers	Our answers
2	Reviewer #2: Yoshimura and colleagues describe the in vitro quantification of alum-adsorbed P. multocida toxin (PMT). In principle, the manuscript addresses an important issue and the experiments follow a clear rationale. However, the authors should demonstrate the quantitative desorption of antigens. It remains unclear, how much antigen is still retained with the alum. In addition, it seems that all experiments have been performed once and without replicates. So, there is no statistical evaluation of the data. The broad body of literature on antigen desorption should be considered and appropriately cited.	Thank you for the summary of our paper. Let us correct one point: this paper proposes dot blotting as a method that does not require desorption of antigens. Therefore, we consider that quantitative determination of desorption is unnecessary.<br><br>On the other hand, as you pointed out, each experiment was not repeated, and statistical analyses were lacking. This time, we conducted additional experiments, performed statistical analyses on each experimental result, and made corrections. Would you confirm the revised Results section?	Antigen that is adsorbed to the alum matrix is not accessible to immune detection by dot blotting, but is immunologically active. If the assay is meant to measure the antigen content IT IS NECESSARY to quantitatively determine the amount of antigen. If the authors refuse to provide evidence for this, the paper is not suited for publication.<br><br>Showing an error bar is no statistical analysis.<br><br>Why has the comment regarding the broad body of literature been ignored?
3	Line 86 ff. – adsorption of PMT on aluminium hydroxide<br><br>- The process of alum adsorption is relatively fast, but the duration of the adsorption process has an influence on the strength of binding. An over night adsorption might not be representative for commercial products.	As you pointed out, it is possible that the adsorption of the antigen onto the aluminum gel is completed without waiting overnight. The overnight reaction step is thought to be due to efficiencies in the vaccine manufacturing process. In this paper, we prepared each sample according to the requirements for vaccine approval and aimed to quantify the amount of antigen in the vaccine.	The reviewer’s comment has not been answered. The adsorption process continues during storage. An overnight adsorption is too short.

4	Usually, antigens -such as tetanus toxoids- are formalin treated before alum adsorption. What is the rationale for the fixation step after the adsorption?	As you pointed out, toxoids are usually "inactivated" with formalin beforehand because the antigenic toxins have pathogenicity. However, as we answered to Editor's comment No.2, the antigens of the AR and AR/ER vaccines discussed in this paper are recombinant non-toxic<br><br>mutant toxins, so an "inactivation" step is unnecessary. The main purpose of formalin treatment after aluminum gel adsorption is "fixation." In fact, the need for this "fixation" treatment is not scientifically justified, but the samples were prepared in a manner similar to vaccine production, in accordance with veterinary drug approvals.	It is not convincing to read that there was no scientific rationale for the fixation treatment. For vaccine production formalin fixation is done before alum adsorption. The process described by the authors is not comparable.
5	Line 101 – Neutralizing activity of anti PMT MAb: Please provide a reference.	Unfortunately, there are no published papers on the neutralizing titers of this antibody. As a result of in-house testing in the past following the method described on page 8, line 128, it was confirmed that this MAb has 256 times more neutralizing capacity. We have added the neutralizing antibody titers on page 6, line 104.	Line 108 is a head line, monoclonal antibodies are not mentioned.
6	Line 106 – Dot blotting<br><br>- Desorption of antigens from aluminum hydroxide salts is achieved by high ionic strength and/or high pH of the desorption buffer (as alum reaches neutral charge at ~pH 9). In addition, the process is time dependent. The process described in the manuscript uses neither of these mechanisms. It is hard to conceive that quantitative desorption was reached. This should be demonstrated, also with commercial vaccine batches.	The dot blotting method described in this paper is proposed as a direct quantitative method for the antigen-adjuvant complex that does not require the desorption of the antigen from the aluminum gel. Therefore, we believe that treatment with high ionic strength or high pH is unnecessary in this test system.	The personal conviction of the authors is irrelevant. They were requested to demonstrate quantitative desorption of the antigens.
7	Line 188 – Dot blotting Results Fig 1 & 2	As answered in No. 6, we believe that desorption treatment is unnecessary. On the other hand, as you	To insert error bars is no statistical analysis.

	- Desorption should be performed in replicates. Results should be statistically analyzed.	pointed out, statistical analysis was lacking, so we performed additional experiments and statistical analysis, corrected Fig 1, 2, Table 1, S1, S2, S4 and added Table S5. The text has been revised due to additional experiments as follows. Materials and methods section: page 7, line 111<br><br>Results section: page 11, line 183, 187 to 191, Fig 1 footnote, page 12, line 205, 209 to 213, Fig 2 footnote, page 15, line 250 to 252, Table 1 footnote	
8	Line 202 ff. – Adsorption using different concentrations of Alum<br><br>- It is surprising to see non-adsorbed antigen with the highest alum concentration. Is there an explanation, why the antigen content decreases from 10 to 7.5 mg/ml Alum, although the amount of free antigen seems to be lowest with 7.5 mg/ml?	As you pointed out in No. 2 and No. 7, the error was due to the lack of the number of replicates of the experiment. Multiple experiments have been repeated and it has been shown that the amount of free antigen in the supernatant is dependent on the concentration of aluminum gel. We would appreciate your confirmation of the revised Fig 2.	Why did the authors not think of this possibility in the first place?.
9	Line 228 – You probably refer to "raw" data?	We apologize for the human error. We have modified it.	
10	Line 248 ff. – The PMT content of AR and AR/ER vaccines<br><br>- Is there data available, whether other antigens, such as ER, influence the detection of PMT?	We have added supplemental data (Fig S1) comparing the reactivity (specificity) of MAbs against AR, AR/ER vaccines, and each antigen (page 16, line 279).	The question was, whether ER interferes with the quantitative detection of PMT antigens, e.g. by reducing the binding capacity of the nitrocellulose membrane. This question was not addressed.<br><br>If this is a PMT-specific mAb, why is there reactivity with ER antigens?.
11	- What is the 95% confidence interval of the antibody titres? Are the titres significantly different?	Statistical analysis of neutralizing antibody titers has been added to Table S4. The difference in geometric mean values were observed, but no statistically significant differences were observed due to individual dispersion.	What do the authors conclude from the figure, if the data is not significant and does not show any trend?
