# Supplementary material for: Method for quantifying the Pasteurella multocida antigen adsorbed on aluminum hydroxide adjuvant in swine atrophic rhinitis vaccine
Source: PLoS One. 2024 May 20;19(5):e0301688. doi: 10.1371/journal.pone.0301688 (PMC11104628; doi:10.1371/journal.pone.0301688)
Supplement: Attachment — Submitted filename: Response to Reviewers.docx [file pone.0301688.s008.docx]

Response Letter

Paulo Lee Ho, Ph.D.

Academic Editor

PLOS ONE

7^th^ September 2023

Dear Dr. Paulo Lee Ho

Subject: Ms. Ref. No. PONE-D-23-12736
Title: Method for quantifying the Pasteurella multocida antigen adsorbed on aluminum hydroxide adjuvant in swine atrophic rhinitis vaccine

Thank you for your email dated 20^th^ July 2023 enclosing the reviewer’s comments to our paper PONE-D-23-12736.

We have carefully reviewed the comments and have revised the manuscript accordingly. Our responses are given in a point-by-point manner below. Changes to the manuscript are shown in the revised version with track changes. The page and line number (i.e. page X, line Y) has been reset with the revisions. Thank you in advance for your understanding and acceptance.

We hope the revised version will be now suitable for publication in PLOS ONE	.

Please let me know if you have any questions or concerns.

Sincerely,

Masaya Yoshimura

Response to the Editor:

We appreciate helpful suggestions of the Editor. Our responses to your suggestions are below.

| No. | Your comment | Our answer |
| --- | --- | --- |
| 1 | Provide experimental data showing how much (antigen) is bound in alum. | From the results in Fig 1 and especially Fig 2, when the antigen concentration and aluminum concentration were optimal (in this study 500 μg/mL and 10 mg/mL, respectively), there was almost no antigen remaining in the supernatant. Therefore, we consider that almost all of the antigen is adsorbed to the aluminum adjuvant, as described in the text on page 13, line 228. |
| 2 | Provide a rational for the inactivation process after adsorption of the antigen to alum. This is not usual. In general, the antigen is treated with formaldehyde and then adsorbed on alum. | Preliminary "inactivation" with formalin is necessary in the case of toxoids whose antigenic toxins have pathogenicity. Since the antigens of the AR and AR/ER vaccines discussed in this paper are recombinant non-toxic mutant toxins, we believe that an "inactivation" step is unnecessary.  The main purpose of formalin treatment after aluminum gel adsorption is "fixation." In fact, the need for this "fixation" treatment is not scientifically justified, but the samples were prepared in a manner similar to vaccine production, in accordance with veterinary drug approvals. |
| 3 | Please, answer to all the comments raised by both reviewers. | We carefully considered the reviewers' comments and revised the manuscript accordingly. As shown in the table below, we have sincerely responded to each of the points raised. |

Response to Reviewers:

Thank you for your review of our paper. Our answers to your comments are as follows.

| No. | Your comments | Our answers |
| --- | --- | --- |
| 1 | Reviewer #1: I am concerned about how the toxin was bound to the adjuvant since Phosphate interferes and was included in all mixing and testing buffers. Because of the including of the phosphate buffer I don't agree with the assumption of 100% bound. No measurements were made to evaluate the amount of toxin bound and no evaluation was done to look at parameters to increase binding (time and buffer). This is a significant gap to the work conducted. | We agree that phosphate buffer may interfere with the binding of antigen to aluminum gel. Therefore, we believe that current antigen quantification methods, which are based on the amount of antigen measured before adsorption to aluminum gel and approximated by assuming 100% binding, are somewhat problematic. In other words, in this paper we would like to emphasize the usefulness of dot blotting for quality assurance, which allows us to directly quantify the amount of antigen in the final product. The manufacturing method (such as reaction time and buffer solution, etc.) was studied without any change from the veterinary drug approvals, so it was not evaluated in this paper. |
| 2 | Reviewer #2: Yoshimura and colleagues describe the in vitro quantification of alum-adsorbed P. multocida toxin (PMT). In principle, the manuscript addresses an important issue and the experiments follow a clear rationale. However, the authors should demonstrate the quantitative desorption of antigens. It remains unclear, how much antigen is still retained with the alum. In addition, it seems that all experiments have been performed once and without replicates. So, there is no statistical evaluation of the data. The broad body of literature on antigen desorption should be considered and appropriately cited. | Thank you for the summary of our paper.  Let us correct one point: this paper proposes dot blotting as a method that does not require desorption of antigens. Therefore, we consider that quantitative determination of desorption is unnecessary.  On the other hand, as you pointed out, each experiment was not repeated, and statistical analyses were lacking. This time, we conducted additional experiments, performed statistical analyses on each experimental result, and made corrections. Would you confirm the revised Results section? |
| 3 | Line 86 ff. – adsorption of PMT on aluminium hydroxide  - The process of alum adsorption is relatively fast, but the duration of the adsorption process has an influence on the strength of binding. An over night adsorption might not be representative for commercial products. | As you pointed out, it is possible that the adsorption of the antigen onto the aluminum gel is completed without waiting overnight. The overnight reaction step is thought to be due to efficiencies in the vaccine manufacturing process. In this paper, we prepared each sample according to the requirements for vaccine approval and aimed to quantify the amount of antigen in the vaccine. |
| 4 | Usually, antigens -such as tetanus toxoids- are formalin treated before alum adsorption. What is the rationale for the fixation step after the adsorption? | As you pointed out, toxoids are usually "inactivated'' with formalin beforehand because the antigenic toxins have pathogenicity. However, as we answered to Editor's comment No.2, the antigens of the AR and AR/ER vaccines discussed in this paper are recombinant non-toxic mutant toxins, so an "inactivation" step is unnecessary. The main purpose of formalin treatment after aluminum gel adsorption is "fixation." In fact, the need for this "fixation" treatment is not scientifically justified, but the samples were prepared in a manner similar to vaccine production, in accordance with veterinary drug approvals. |
| 5 | Line 101 – Neutralizing activity of anti PMT MAb: Please provide a reference. | Unfortunately, there are no published papers on the neutralizing titers of this antibody. As a result of in-house testing in the past following the method described on page 8, line 128, it was confirmed that this MAb has 256 times more neutralizing capacity. We have added the neutralizing antibody titers on page 6, line 104. |
| 6 | Line 106 – Dot blotting  - Desorption of antigens from aluminum hydroxide salts is achieved by high ionic strength and/or high pH of the desorption buffer (as alum reaches neutral charge at ~pH 9). In addition, the process is time dependent. The process described in the manuscript uses neither of these mechanisms. It is hard to conceive that quantitative desorption was reached. This should be demonstrated, also with commercial vaccine batches. | The dot blotting method described in this paper is proposed as a direct quantitative method for the antigen-adjuvant complex that does not require the desorption of the antigen from the aluminum gel. Therefore, we believe that treatment with high ionic strength or high pH is unnecessary in this test system. |
| 7 | Line 188 – Dot blotting Results Fig 1 & 2  - Desorption should be performed in replicates. Results should be statistically analyzed. | As answered in No. 6, we believe that desorption treatment is unnecessary. On the other hand, as you pointed out, statistical analysis was lacking, so we performed additional experiments and statistical analysis, corrected Fig 1, 2, Table 1, S1, S2, S4 and added Table S5.  The text has been revised due to additional experiments as follows.  Materials and methods section: page 7, line 111  Results section: page 11, line 183, 187 to 191, Fig 1 footnote, page 12, line 205, 209 to 213, Fig 2 footnote, page 15, line 250 to 252, Table 1 footnote |
| 8 | Line 202 ff. – Adsorption using different concentrations of Alum  - It is surprising to see non-adsorbed antigen with the highest alum concentration. Is there an explanation, why the antigen content decreases from 10 to 7.5 mg/ml Alum, although the amount of free antigen seems to be lowest with 7.5 mg/ml? | As you pointed out in No. 2 and No. 7, the error was due to the lack of the number of replicates of the experiment. Multiple experiments have been repeated and it has been shown that the amount of free antigen in the supernatant is dependent on the concentration of aluminum gel. We would appreciate your confirmation of the revised Fig 2. |
| 9 | Line 228 – You probably refer to “raw” data? | We apologize for the human error. We have modified it. |
| 10 | Line 248 ff. – The PMT content of AR and AR/ER vaccines  - Is there data available, whether other antigens, such as ER, influence the detection of PMT? | We have added supplemental data (Fig S1) comparing the reactivity (specificity) of MAbs against AR, AR/ER vaccines, and each antigen (page 16, line 279). |
| 11 | - What is the 95% confidence interval of the antibody titres? Are the titres significantly different? | Statistical analysis of neutralizing antibody titers has been added to Table S4. The difference in geometric mean values were observed, but no statistically significant differences were observed due to individual dispersion. |

Response to Reviewers’ comments in manuscript

| No. | Your comments | Our answers |
| --- | --- | --- |
| 1 | Line 24.  This is not defined before. I assume this is the protective toxin of P. multocida. | As suggested, we have changed the word from dermonecrotic to protective. |
| 2 | Line 88: Phosphate buffer can interfere with antigen binding to alum. Were different phosphate concentrations or alternate buffers tested? | Thank you for your comment. Indeed, we agree that phosphate buffers can interfere with antigen binding to alum. However, as shown in the result in Fig 2, when the antigen concentration and aluminum concentration were optimal, there was almost no antigen remaining in the supernatant. Therefore, we considered that PBS with the composition used in this study has less inhibitory activity. In addition, in this paper, the antigen preparation was performed in according to the method described in the veterinary drug approval documents for AR and AR/ER vaccines, so no tests were conducted with other buffers. In the Materials and Methods section, we clearly stated this (page 5, line 77, page 6, line 89). |
| 3 | Line 88: Shouldn't these be defined concentrations of antigen and aluminum before mixing? | Thank you for your suggestion.  The word "arbitrary" was inappropriate. As described in the results section, we have prepared and tested multiple concentrations, so we have revised the description to "different" (page 6, line 92). |
| 4 | Line 90: What is the purpose of fixing the solution after adjuvanting? | As answered to Editor's comment No.2, the need for this "fixation" treatment is not scientifically justified, but the samples were prepared in a manner similar to vaccine production, in accordance with veterinary drug approvals. |
| 5 | Line 93: Does washing with PBS displace the bound antigen? Was Tris buffer considered? | As you pointed out, when washing with PBS, the possibility of desorption of antigens from the alum cannot be excluded. However, since sufficient intensities were confirmed in this dot blotting study and an immune response in piglets was also confirmed, we concluded that desorption is at a negligible level. Also, as answered in No.2, we did not test other buffers. |
| 6 | Line 107: Are the test samples stable in PBS? This is likely to lead to desorbtion of the antigen. | For the same reason as in answer No. 5, we think there is no problem using PBS as the dilution solvent. |
| 7 | Line 186: How was free and bound toxin measured? Were different adsorption times evaluated. Were samples continually mixed overnight during the 2-8C incubation? | In the paragraph you refer to (Fig 1), free antigen was not measured. We only measured it in the study for Fig 2. Regarding the adsorption time, although there is a difference of several hours, it has been unified as overnight. The Materials and Methods wording was revised because the incubation status was static (page 6, line 93 and 96). |
| 8 | Line 280: This should be evaluated by measuring the protein content in the supernatant. It is likely not 100% especially as it is exposed to phosphate buffer which interferes with binding. | Let us explain to clear up any misunderstandings. This sentence states that the amount of antigen in the final product should be directly measured as it may not be 100% binding as you pointed out. |
| 9 | Line281. Have different alum concentrations been evaluated for efficacy with this vaccine candidate? What is the target alum and toxin dose? | A commercially available aluminum gel (10 mg/mL) was typically used, and no concentration dependence evaluation of the aluminum gel was performed in the efficacy study. The target input amount for toxins is set at 40 µg/mL. |
| 10 | Line 289: SDS-PAGE is not a quantitative method | Let us clear up some misconceptions: SDS-PAGE is not used to quantify antigens adsorbed on aluminum gels. As described on page 5, line 83, the antigen was processed by SDS-PAGE prior to adsorption and quantified using bovine serum albumin as a standard. |
| 11 | Line 289: samples containing alum are also not able to enter into the gel. |  |

Again, thank you for giving us the opportunity to strengthen our manuscript with your valuable comments and queries. We have worked hard to incorporate your feedback and hope that these revisions persuade you to accept our submission.

Sincerely,

Masaya Yoshimura
