# Supplementary material for: Method for quantifying the Pasteurella multocida antigen adsorbed on aluminum hydroxide adjuvant in swine atrophic rhinitis vaccine
Source: PLoS One. 2024 May 20;19(5):e0301688. doi: 10.1371/journal.pone.0301688 (PMC11104628; doi:10.1371/journal.pone.0301688)
Supplement: Attachment — Submitted filename: Rebuttal letter.docx [file pone.0301688.s010.docx]

Rebuttal Letter

Paulo Lee Ho, Ph.D.

Academic Editor

PLOS ONE

13^th^ November 2023

Dear Dr. Paulo Lee Ho

Subject: Ms. Ref. No. PONE-D-23-12736R1
Title: Method for quantifying the Pasteurella multocida antigen adsorbed on aluminum hydroxide adjuvant in swine atrophic rhinitis vaccine

Thank you for your re-reviewing of our revised version. On November 9th, I received an email from the publishing editor, Dr. Emaan Basat. Following her recommendation, we will resubmit this rebuttal letter so that you, the academic editor, can accurately evaluate our manuscript.

Could you please review the gist of this paper and decide if corrections are necessary in response to Reviewer 2's points? Please let me know if you have any questions or concerns.

Sincerely,

Masaya Yoshimura

Response to the Editor:

Thank you for your email of October 19 in response to our inquiry. We would like to describe our interpretation of that email.

| No. | Dr. Paulo Lee Ho’s comment | Our answer |
| --- | --- | --- |
| 1 | Dear Prof. Masaya Yoshimura,  Thank you very much for your kind message. I would suggest you proceed with the responses to all the questions. I will analyse all the comments and decide based on the arguments from both sides. I can not decide right now based on what you wrote below. I will need to read the manuscript, the revised manuscript, the reviewer´s comments, your rebuttal as well as the reviewer´s response on your rebuttal. After following all these steps I will be able to decide on the matter.  Best wishes, | You mentioned that "I cannot decide right away, and I will need to read the manuscript, its revisions, etc.".  Therefore, we have been waiting for your confirmation and decision.  We are sending you this rebuttal letter to assist you in making a decision. We would appreciate it if you could review it. |

Response to Reviewer 2:

| No. | Your comments | Our answers | Your comments 2 | Our answers 2 |
| --- | --- | --- | --- | --- |
| 2 | Reviewer #2: Yoshimura and colleagues describe the in vitro quantification of alum-adsorbed P. multocida toxin (PMT). In principle, the manuscript addresses an important issue and the experiments follow a clear rationale. However, the authors should demonstrate the quantitative desorption of antigens. It remains unclear, how much antigen is still retained with the alum. In addition, it seems that all experiments have been performed once and without replicates. So, there is no statistical evaluation of the data. The broad body of literature on antigen desorption should be considered and appropriately cited. | Thank you for the summary of our paper.  Let us correct one point: this paper proposes dot blotting as a method that does not require desorption of antigens. Therefore, we consider that quantitative determination of desorption is unnecessary.  On the other hand, as you pointed out, each experiment was not repeated, and statistical analyses were lacking. This time, we conducted additional experiments, performed statistical analyses on each experimental result, and made corrections. Would you confirm the revised Results section? | Antigen that is adsorbed to the alum matrix is not accessible to immune detection by dot blotting, but is immunologically active. If the assay is meant to measure the antigen content IT IS NECESSARY to quantitatively determine the amount of antigen. If the authors refuse to provide evidence for this, the paper is not suited for publication.  Showing an error bar is no statistical analysis.  Why has the comment regarding the broad body of literature been ignored? | Reviewer 2 states that "antigen that adsorbed to the alum matrix is not accessible to immune detection by dot blotting", but this is a major misunderstanding of what our paper claims.  We cannot understand what kind of literature we should search due to this misunderstanding, therefore the request of reviewer 2 has not been supported yet.  Regarding statistical processing, we believe that displaying error bars is one of the statistical processing methods that shows the dispersion of multiple test results. In addition, for procedures such as t-tests, we have already included the results in the supplemental table for those that were determined to be meaningful. |
| 3 | Line 86 ff. – adsorption of PMT on aluminium hydroxide  - The process of alum adsorption is relatively fast, but the duration of the adsorption process has an influence on the strength of binding. An over night adsorption might not be representative for commercial products. | As you pointed out, it is possible that the adsorption of the antigen onto the aluminum gel is completed without waiting overnight. The overnight reaction step is thought to be due to efficiencies in the vaccine manufacturing process. In this paper, we prepared each sample according to the requirements for vaccine approval and aimed to quantify the amount of antigen in the vaccine. | The reviewer’s comment has not been answered. The adsorption process continues during storage. An overnight adsorption is too short. | We do not agree with the suggestion that the reaction time is too short because some products go to the next step immediately after adsorbing the antigen and aluminum gel.  If we considered the reaction during the storage of the product, we would have to track it for more than two years, which we think is nonsense. |
| 4 | Usually, antigens -such as tetanus toxoids- are formalin treated before alum adsorption. What is the rationale for the fixation step after the adsorption? | As you pointed out, toxoids are usually "inactivated'' with formalin beforehand because the antigenic toxins have pathogenicity. However, as we answered to Editor's comment No.2, the antigens of the AR and AR/ER vaccines discussed in this paper are recombinant non-toxic mutant toxins, so an "inactivation" step is unnecessary. The main purpose of formalin treatment after aluminum gel adsorption is "fixation." In fact, the need for this "fixation" treatment is not scientifically justified, but the samples were prepared in a manner similar to vaccine production, in accordance with veterinary drug approvals. | It is not convincing to read that there was no scientific rationale for the fixation treatment. For vaccine production formalin fixation is done before alum adsorption. The process described by the authors is not comparable. | In this paper, we attempt to quantify antigens using the same method as the manufacturing method of the product. The reviewer's comments are "suggestions on the manufacturing method", but this point is a different issue from the purpose of this paper. It is not easy to change a process that has already been approved, and that's not what we want to do. |
| 5 | Line 101 – Neutralizing activity of anti PMT MAb: Please provide a reference. | Unfortunately, there are no published papers on the neutralizing titers of this antibody. As a result of in-house testing in the past following the method described on page 8, line 128, it was confirmed that this MAb has 256 times more neutralizing capacity. We have added the neutralizing antibody titers on page 6, line 104. | Line 108 is a head line, monoclonal antibodies are not mentioned. | Is the item you are referring to on line 128?  We listed a heading that describes how to measure neutralizing antibody titers. The specific method is described on page 8, line 130 to page 9, line 148. |
| 6 | Line 106 – Dot blotting  - Desorption of antigens from aluminum hydroxide salts is achieved by high ionic strength and/or high pH of the desorption buffer (as alum reaches neutral charge at ~pH 9). In addition, the process is time dependent. The process described in the manuscript uses neither of these mechanisms. It is hard to conceive that quantitative desorption was reached. This should be demonstrated, also with commercial vaccine batches. | The dot blotting method described in this paper is proposed as a direct quantitative method for the antigen-adjuvant complex that does not require the desorption of the antigen from the aluminum gel. Therefore, we believe that treatment with high ionic strength or high pH is unnecessary in this test system. | The personal conviction of the authors is irrelevant. They were requested to demonstrate quantitative desorption of the antigens. | Reviewer 2 states that "high ionic strength and/or high pH affect the desorption of antigens from alum gel". Yes, we agree. However, our dot blotting method does not require the desorption of the antigen from the aluminum gel, and the antigens in this test system and the vaccine are prepared under the same conditions.  Therefore, consideration of these conditions is unnecessary in this test system. |
| 7 | Line 188 – Dot blotting Results Fig 1 & 2  - Desorption should be performed in replicates. Results should be statistically analyzed. | As answered in No. 6, we believe that desorption treatment is unnecessary. On the other hand, as you pointed out, statistical analysis was lacking, so we performed additional experiments and statistical analysis, corrected Fig 1, 2, Table 1, S1, S2, S4 and added Table S5.  The text has been revised due to additional experiments as follows.  Materials and methods section: page 7, line 111  Results section: page 11, line 183, 187 to 191, Fig 1 footnote, page 12, line 205, 209 to 213, Fig 2 footnote, page 15, line 250 to 252, Table 1 footnote | To insert error bars is no statistical analysis. | As we answered in comment No. 2, displaying error bars is one of the statistical processing methods that shows the dispersion of multiple test results. In addition, we have already included the t-test results in the supplemental table. |
| 8 | Line 202 ff. – Adsorption using different concentrations of Alum  - It is surprising to see non-adsorbed antigen with the highest alum concentration. Is there an explanation, why the antigen content decreases from 10 to 7.5 mg/ml Alum, although the amount of free antigen seems to be lowest with 7.5 mg/ml? | As you pointed out in No. 2 and No. 7, the error was due to the lack of the number of replicates of the experiment. Multiple experiments have been repeated and it has been shown that the amount of free antigen in the supernatant is dependent on the concentration of aluminum gel. We would appreciate your confirmation of the revised Fig 2. | Why did the authors not think of this possibility in the first place?. | I am sorry. |
| 9 | Line 228 – You probably refer to “raw” data? | We apologize for the human error. We have modified it. |  |  |
| 10 | Line 248 ff. – The PMT content of AR and AR/ER vaccines  - Is there data available, whether other antigens, such as ER, influence the detection of PMT? | We have added supplemental data (Fig S1) comparing the reactivity (specificity) of MAbs against AR, AR/ER vaccines, and each antigen (page 16, line 279). | The question was, whether ER interferes with the quantitative detection of PMT antigens, e.g. by reducing the binding capacity of the nitrocellulose membrane. This question was not addressed. If this is a PMT-specific mAb, why is there reactivity with ER antigens?. | We judge the response to ER to be negative based on the results in Fig. S1. |
| 11 | - What is the 95% confidence interval of the antibody titres? Are the titres significantly different? | Statistical analysis of neutralizing antibody titers has been added to Table S4. The difference in geometric mean values were observed, but no statistically significant differences were observed due to individual dispersion. | What do the authors conclude from the figure, if the data is not significant and does not show any trend? | Although there is no significant difference, but it has been suggested that a small amount of antigen in a vaccine may affect its efficacy (ability to induce neutralizing antibodies).  We hope that you understand that unlike small animals such as mice, the number of animals that can be used is limited. |
