# Supplementary material for: Method for quantifying the Pasteurella multocida antigen adsorbed on aluminum hydroxide adjuvant in swine atrophic rhinitis vaccine
Source: PLoS One. 2024 May 20;19(5):e0301688. doi: 10.1371/journal.pone.0301688 (PMC11104628; doi:10.1371/journal.pone.0301688)
Supplement: Attachment — Submitted filename: Response to Reviewers.docx [file pone.0301688.s011.docx]

Response Letter

Paulo Lee Ho, Ph.D.

Academic Editor

PLOS ONE

14^th^ February 2024

Dear Dr. Paulo Lee Ho

Subject: Ms. Ref. No. PONE-D-23-12736R2
Title: Method for quantifying the *Pasteurella multocida* antigen adsorbed on aluminum hydroxide adjuvant in swine atrophic rhinitis vaccine

Thank you for your email of December 21, 2023, with the reviewer's comments on our paper PONE-D-23-12736.

We have carefully reviewed the comments and revised the manuscript accordingly. Our responses are provided below on a point-by-point basis. Changes to the manuscript are indicated in the revised version with track changes. The page and line numbers (i.e. page X, line Y) have been reset with the revisions. We thank you in advance for your understanding and acceptance.

We hope that the revised version is now suitable for publication in PLOS ONE.

Please let me know if you have any questions or concerns.

Sincerely,

Masaya Yoshimura

Response to the Editor:

Thank you for your very kind response to our rebuttal submitted on November 13, 2023.

We are grateful to have found a third-party reviewer.

Corrections have been made in response to the reviewer's comments. We hope that this revised version is acceptable for publication.

Response to Reviewer:

Thank you for your review of our paper. Our answers to your comments are as follows.

| No. | Your comments | Our answers |
| --- | --- | --- |
| 1 | The manuscript is a good attempt. The desorption of antigens from Aluminum hydroxide adjuvant without disturbing the antigenicity for ELISA methods is always a challenge. The authors report the use of dot blot assay for quantification of antigens. Dot blot assay is a semiquantitative method. The authors report its use for quantitative use for gel adsorbed vaccines. The data is good; however, needs a very convincing and robust discussion in the manuscript on following. | Thank you for your understanding of our proposed antigen quantification using a dot blot assay. We have performed additional experiments and revised the manuscript based on the points you have raised below. Could you please confirm the revised part? |
| 2 | 1. The data should be compared to method in prior art which is quantitative in function | As described on page 5, lines 83 to 86, this antigen was conventionally quantified by SDS-PAGE using BSA as a standard.  As you pointed out, we confirmed the results of the SDS-PAGE. As predicted, PMT bound to aluminum hydroxide could not be electrophoresed even after SDS treatment. Therefore, we considered eluting PMT using an extraction method with increased ionic strength, but the extraction efficiency was low. Comparison with dot blotting confirmed the superiority of the dot blot assay, which does not require desorption with aluminum hydroxide and can detect with higher sensitivity.  SDS-PAGE and PMT extraction methods from aluminum hydroxide are described on page 7, lines 110 to 116 and page 7, lines 118 to 124, respectively.  Additional assay results and discussion are provided on page 14, lines 239 to 249 and page 18, lines 298 to 308, respectively. |
| 3 | 2. The data set should also include robust method validation data as per ICH guidelines on quantitative methods with focus on recovery and precision | As described on page 15, lines 255 to 258, we have established a dot blot assay system using PMT-alum as the standard antigen and performed validation of the system according to your comment. The results are shown in Table 2, and detailed analysis information is shown in Supplementary Table 4.  The validation focused on four points: recovery, precision, lower limit of quantification, and linearity. As discussed on page 21, lines 346 to 348, further evaluation may be required before it can be established as a quality test.  In addition, the dot blot assay system has been improved, so we re-measured the amount of antigen in the vaccine. Although the results did not change significantly, we have updated Table 3. |
| 4 | 3. The manuscript should include a detailed development account on how desorption conditions were designed to get a good recovery. | As answered in comment No. 2, we have desorbed PMT from aluminum hydroxide. On the other hand, the dot blotting method described in this paper is proposed as a direct method for quantification of antigen-adjuvant complexes that does not require desorption of antigen from aluminum gel. Therefore, we did not conduct an in-depth study of the desorption conditions that showed a good recovery rate. |
| 5 | 4. Dot blot method LOQ and how it will be used in vaccine testing should be stressed upon in discussion. | Thank you for your comment. We agree with your point. The amount of antigen in the vaccine was well within the quantification range of the assay system set up this time. We have clearly stated this in the Discussion section (page 20, lines 340 to 342). |

Thank you again for giving us the opportunity to strengthen our manuscript with your valuable comments. We have worked hard to incorporate your feedback and hope that these revisions persuade you to accept our submission.

Sincerely,

Masaya Yoshimura
